# Short-term vaginal prasterone therapy induces steroid receptor modulation and extracellular matrix remodeling in human vaginal mucosa

**DOI:** 10.1038/s41598-026-50491-5

**Published:** 2026-05-19

**Authors:** Katarzyna Tomczyk, K. C. Yang-Jensen, L. G. Lorentzen, C. Y. Chuang, M. J. Davies, M. Kampioni, K. Chmaj-Wierzchowska, M. Wilczak, M. Kędzia

**Affiliations:** 1 Department of Reproduction and Gynecology, Gynecologic and Obstetrical University Hospital, Poznan University of Medical Sciences, Poznan, Poland; 2 Department of Biomedical Sciences, Panum Institute, University of Copenhagen, Copenhagen, Denmark; 3 Department of Vascular Surgery, Heart Center, University Hospital Copenhagen - Rigshospitalet, Copenhagen, Denmark; 4 Department of Mother’s and Child’s Health and Minimally Invasive Gynecology, Gynecologic and Obstetrical University Hospital, Poznan University of Medical Sciences, Poznan, Poland

**Keywords:** Prasterone, Collagen IV, GSM, Urogynecology, Cell biology, Diseases, Medical research

## Abstract

Prasterone (dehydroepiandrosterone, DHEA) is increasingly used in the treatment of genitourinary syndrome of menopause (GSM), although its molecular effects on vaginal tissue remain insufficiently characterized. This prospective study evaluated the short-term impact of vaginal prasterone on steroid receptor expression, epithelial morphology, extracellular matrix composition, and the proteomic profile of the vaginal mucosa. Ten women aged 41–67 years received intravaginal prasterone (6.5 mg daily) for eight weeks. Vaginal biopsies were obtained before and after treatment, and serum estradiol and DHEA-S levels were assessed. Immunohistochemical analysis demonstrated a significant increase in cumulative ERα expression across epithelial layers (*p* = 0.0379), with the most pronounced changes in the superficial epithelium and stromal compartment. Progesterone receptor expression showed stromal upregulation accompanied by epithelial downregulation. Epithelial thickness increased by 17.5 μm following therapy. Proteomic analysis using LC–MS/MS identified 87 proteins with altered abundance, including enrichment of extracellular matrix components and upregulation of collagen IV (COL4A1 and COL4A2). In contrast, several inflammation- and remodeling-related proteins, including IL18, LCN2, SERPINE2, and PRSS27, were downregulated. These findings indicate that short-term vaginal prasterone therapy promotes steroid receptor modulation and extracellular matrix remodeling in human vaginal mucosa while reducing inflammatory signaling pathways. Patient-reported outcomes assessed using the WHOQOL-BREF questionnaire indicated a trend toward improvement in quality of life following treatment, with the largest median increases observed in the physical (Domain 1) and environmental (Domain 4) domains. However, these changes did not reach statistical significance (*p* > 0.05), and no significant differences were observed in overall quality of life or perceived health status.

**Supplementary Information:**

The online version contains supplementary material available at 10.1038/s41598-026-50491-5.

## Introduction

Genitourinary syndrome of menopause (GSM) is a common condition affecting perimenopausal and postmenopausal women and is characterized by vulvovaginal atrophy, dyspareunia, and urinary symptoms resulting from estrogen deficiency. Local estrogen therapy has long been considered a cornerstone of treatment; however, concerns regarding systemic hormone exposure have stimulated the search for alternative therapies. Prasterone (dehydroepiandrosterone, DHEA), a precursor of both estrogens and androgens, has recently been introduced as a local therapeutic option for GSM. Its pharmacological effect is based on an intracrine mechanism in which DHEA is converted within peripheral tissues into biologically active sex steroids, while only minimal amounts reach the systemic circulation, thereby limiting systemic adverse effects^1^.

Endogenous DHEA is produced mainly by the adrenal glands with a smaller ovarian contribution, and its circulating levels decline markedly with age. Because of this decrease and its role as a precursor of sex steroids, DHEA has been implicated in several age-related physiological processes^2,3^. In menopausal women, local conversion of DHEA into estrogens and androgens within vaginal tissues may partially compensate for hormonal deficiency and contribute to improved epithelial integrity, lubrication, and tissue trophicity. Beyond symptomatic relief in GSM, local hormonal effects may also influence vaginal tissue remodeling, which could be relevant in urogynecological conditions such as stress urinary incontinence or pelvic organ prolapse. Preoperative vaginal estrogenization has been shown to improve mucosal thickness and trophicity, facilitate surgical procedures, and reduce postoperative infection risk through favorable modulation of the vaginal microbiota^4,5^.

The biological effects of estrogens and progesterone are mediated primarily through nuclear receptors. Estrogens act through estrogen receptors ERα and ERβ, whereas progesterone signals via PRA and PRB isoforms^6^. Although DHEA-S itself does not directly bind to nuclear receptors^7^, intracrine metabolism of prasterone may generate active steroids capable of modulating receptor signaling and downstream molecular pathways^1^. Estrogen signaling is known to regulate epithelial proliferation, extracellular matrix composition, and gene expression involved in tissue remodeling and inflammatory responses^8,9^, as well as local aromatase activity influencing estrogen synthesis in peripheral tissues^10,11^.

The aim of this study was therefore to investigate the short-term effects of vaginal prasterone therapy on steroid receptor expression, epithelial morphology, extracellular matrix components, and the proteomic profile of the vaginal mucosa. Using paired vaginal biopsies obtained before and after eight weeks of treatment, we combined immunohistochemical analysis with LC–MS/MS–based proteomics and gene set enrichment analysis to provide mechanistic insight into prasterone-induced tissue remodeling in the human vaginal mucosa.

## Materials and methods

### Study design and participants

This prospective study was conducted at the Department of Mother and Child Health, Gynecological Hospital in Poznań, Poland. Written informed consent was obtained from all participants, and the study protocol was approved by the Ethics Committee of the Poznań University of Medical Sciences (No. 49/20). All methods were performed in accordance with the relevant guidelines and regulations.

**Fig. 1:**
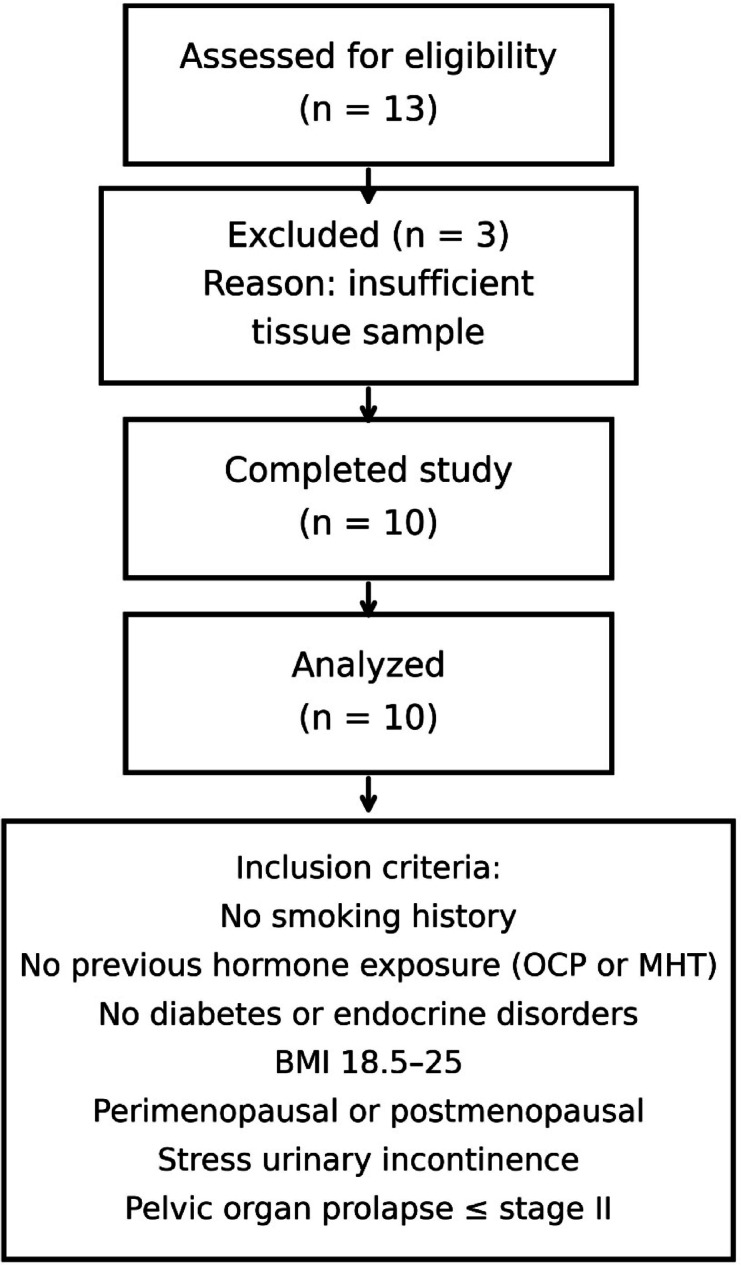
Flow diagram of participant selection and study analyses. Thirteen women were assessed for eligibility, of whom three were excluded due to insufficient tissue samples. Ten participants completed the study and were included in the final analysis.

A total of 10 women aged 41–67 years were enrolled. Inclusion criteria were: (1) diagnosis of either pelvic organ prolapse or stress urinary incontinence, (2) no prior use of vaginal estrogens, and (3) eligibility for surgical treatment. Postmenopausal was defined as ≥ 12 months of amenorrhea. Perimenopausal was defined as menstrual cycle irregularity with preserved ovarian function (Fig. 1).

Blood samples were collected for assessment of estradiol and DHEA-S levels. Serum concentrations were determined using an electrochemiluminescence immunoassay (ECLIA) performed in the hospital’s certified clinical laboratory. Vaginal mucosa biopsies were obtained before and after an eight-week course of daily prasterone (6.5 mg vaginal suppositories; commercially available formulation, Intrarosa^®^). Biopsies were taken from the lower third of the posterior vaginal wall. The first biopsy was performed under local anesthesia with lignocaine, while the second was obtained from the same site (after prasterone application) using the same instrument during urogynecological surgery under general anesthesia. The biopsies included mucosal tissue of approximately 3 mm in depth. Hemostasis was achieved by brief local pressure, and the biopsy sites were left to heal spontaneously without the need for sutures. The procedure was well tolerated, and no significant discomfort or complications were reported.

In perimenopausal participants, biopsies were obtained during the same menstrual cycle phase to minimize hormonal variability. Patient-reported outcome was assessed using the WHOQOL-BREF (World Health Organization Quality of Life – BREF) questionnaire, administered before and after the treatment period.

### Immunohistochemical staining of estrogen and progesterone receptors

Samples, approximately 3 mm in diameter, were immediately immersed in formalin, embedded in paraffin, and sectioned at 0.2 μm thickness. Sections were mounted on adhesive slides and incubated at 60 °C for 60 min, followed by epitope retrieval at 96 °C for 20 min. Staining was performed using the Autostainer Link 48 system (Dako). Estrogen receptor alpha (ERα) was detected with a monoclonal rabbit antibody (clone 1D5), while progesterone receptor (PR) was identified using a monoclonal mouse antibody (clone 1E6). Visualization was achieved with the EnVision Flex+ Mouse, High pH system (Dako). Tissue morphology was examined under an optical microscope (Leica DM3000 equipped with a Flexacam C5 camera).

### Evaluation of receptor expression and tissue morphology

Immunohistochemical evaluation was carried out by one histopathologist and independently verified by a second. The proportion of positively stained cell nuclei was assessed using a semi-quantitative method based on the Allred scoring system. Only distinctly stained nuclei were considered positive and were expressed as a percentage of the total number of cells at 630× magnification. The intensity component of the original Allred score was not applied. Receptor expression was assessed in the lining layer, basal layer of the non-keratinizing epithelium, and superficial layer. In addition, the thickness of the vaginal epithelium was evaluated at 400× magnification, both before and after the treatment period.

### Sample preparation for proteomic (liquid-chromatography-mass spectrometry, LC-MS/MS) analyses

Proteins were extracted and analysed (at the Department of Biomedical Sciences, University of Copenhagen) from tissue samples using the Sample Preparation by Easy Extraction and Digestion (SPEED) method^12^. Following extraction, proteins underwent clean-up, reduction/alkylation and enzymatic digestion with Lys-C (1:100) and trypsin (1:50) as previously described^13^ Digested peptides were subsequently stored at − 80 °C pending LC-MS/MS analysis.

### Liquid chromatography and mass spectrometry

Peptides were resuspended in 0.5% v/v trifluoroacetic acid TFA in water, and separated by liquid chromatography on a Dionex RSLCnano system using a 28 min binary gradient (Solvent A: 0.1% v/v formic acid in water; Solvent B: acetonitrile containing 0.1% v/v formic acid). Eluting peptides were analyzed on a Bruker timsTOF Pro mass spectrometer utilizing data-independent acquisition with parallel accumulation and serial fragmentation (diaPASEF), as previously described by Meier et al.^14^.

### Data processing and statistical analysis

Tissue data analysis was performed using Microsoft Excel 2019 (Microsoft Office), with the serum concentrations of estradiol and DHEA-S, vaginal epithelial thickness, and the presence of estrogen and progesterone receptors before and after treatment, compared using a paired t-test with the Wilcoxon signed-rank test applied; *p* < 0.05 was considered statistically significant.

Pre- and post-treatment WHOQOL-BREF scores were compared using the Wilcoxon signed-rank test. A p-value < 0.05 was considered statistically significant.

For the proteomic data, the raw mass spectrometry files were analyzed using DIA-NN software using a predicted library based on in silico digested protein sequences from the UniProtKB database, with common contaminants included for filtering. Further processing of the data and visualizations was performed using the R statistical programming language. Precursor signals from proteotypic peptides were summarized into protein signals using the MSqRobSum algorithm (as implemented in the msqrob2 package). Protein abundances after treatment were compared to baseline using a robust linear mixed-effects model with patient ID included as a random effect. Proteins were considered differentially abundant if the false discovery rate (FDR)-adjusted (using the Benjamini-Hochberg method) p value was < 0.05^15^.

Gene set enrichment analysis (GSEA) was performed using the clusterProfiler package, with t-statistics from the differential abundance analysis as input. Enrichment was restricted to Gene Ontology (GO) molecular function (MF) terms. GO terms were considered significantly enriched if the FDR-adjusted p-value was < 0.05.

## Results

All laboratory and histopathological findings as well as patient - reported outcomes are summarized in Supplementary Tables 1 and Tables 1 and 2. Serum hormone analysis showed no significant changes following treatment: estradiol levels slightly decreased (41.8 → 38.1pg/ml; p 0.6247), while DHEA-S levels showed a small increase (3.4 → 3.6 µg/dl; p 0.6374).

ERα levels demonstrated an overall increase (43.7% → 62.2%), with the most pronounced changes in the superficial epithelium (10.5% → 19.6%) and stromal layer (8.0% → 12.7%). Although individual layer changes did not reach statistical significance, the cumulative ERα expression across all epithelial layers (ERα B + ERα S + ERα ST) increased significantly after prasterone therapy [4.2 (0.8–5) vs. 15 (0–90); p 0.0379]. In contrast, total PR expression decreased (26.2% → 17.7%), primarily due to reductions in the basal (17.6% → 4.5%) and superficial (1.8% → 0.0%) layers, while stromal PR increased (6.8% → 13.0%). However, the cumulative PR expression did not change significantly [0 (0–80) vs. 1.5 (0–57); p 0.987]. The mean epithelial thickness showed a trend towards an increase (67.75 mm to 85.5 mm) though this did not reach statistical significance (p 0.2309).

Non-parametric analysis using the Wilcoxon signed-rank test confirmed a trend toward improvement in WHOQOL domain scores after treatment. The largest median increase was observed in domain 1 (from 12.0 to 13.5) and domain 4 (from 14.0 to 15.0). However, none of the differences reached statistical significance (*p* > 0.05). No significant changes were observed in the additional items assessing overall quality of life and perceived health status.

**Table 1:** Mean values of serum estradiol and DHEA-S levels, mean percentage of ERα and PR receptor expression and mean epithelial thickness before and after vaginal prasterone treatment.

	Before treatment		After treatment		Change	*p*-value
	Average	Percentage	Average	Percentage		
Estradiol [pg/ml]	41.79		38.07		↓ 3.72	0.6247
DHEA-S [µg/dl]	3.44		3.60		↑ 0.6	0.6374
Basal Erα		25.2%		29.9%	↑ 4.7	0.7038
Superficial Erα		10.53%		19.6%	↑ 9.07	0.3629
Stromal Erα		7.95%		12.65%	↑ 4.7	0.1842
Total Erα		43.68%		62.15%	↑ 18.47	0.0379
Basal PR		17.6%		4.5%	↓ 1.3	0.1314
Superficial PR		1.8%		0.0%	↓ 1.5	0.875
Stromal PR		6.8%		12.97%	↑ 6.7	0.1842
Total PR		26.2%		17.7%	↓ 8.43	0.987
Epithelial thickness [µm]	67.75		85.5		↑ 17.5	0.2309

**Table 2:** Comparison of WHOQOL scores before and after vaginal prasterone treatment.

Variable	Median (pre-treatment)	Median (post-treatment)	*p*-value
Quality of life (item 1)	4.0	4.0	0.78
Self-rated health (item 2)	3.0	3.0	1.00
Domain 1	12.0	13.5	0.33
Domain 2	14.5	14.5	0.72
Domain 3	15.0	15.0	0.63
Domain 4	14.0	15.0	0.38

**Fig. 2:**
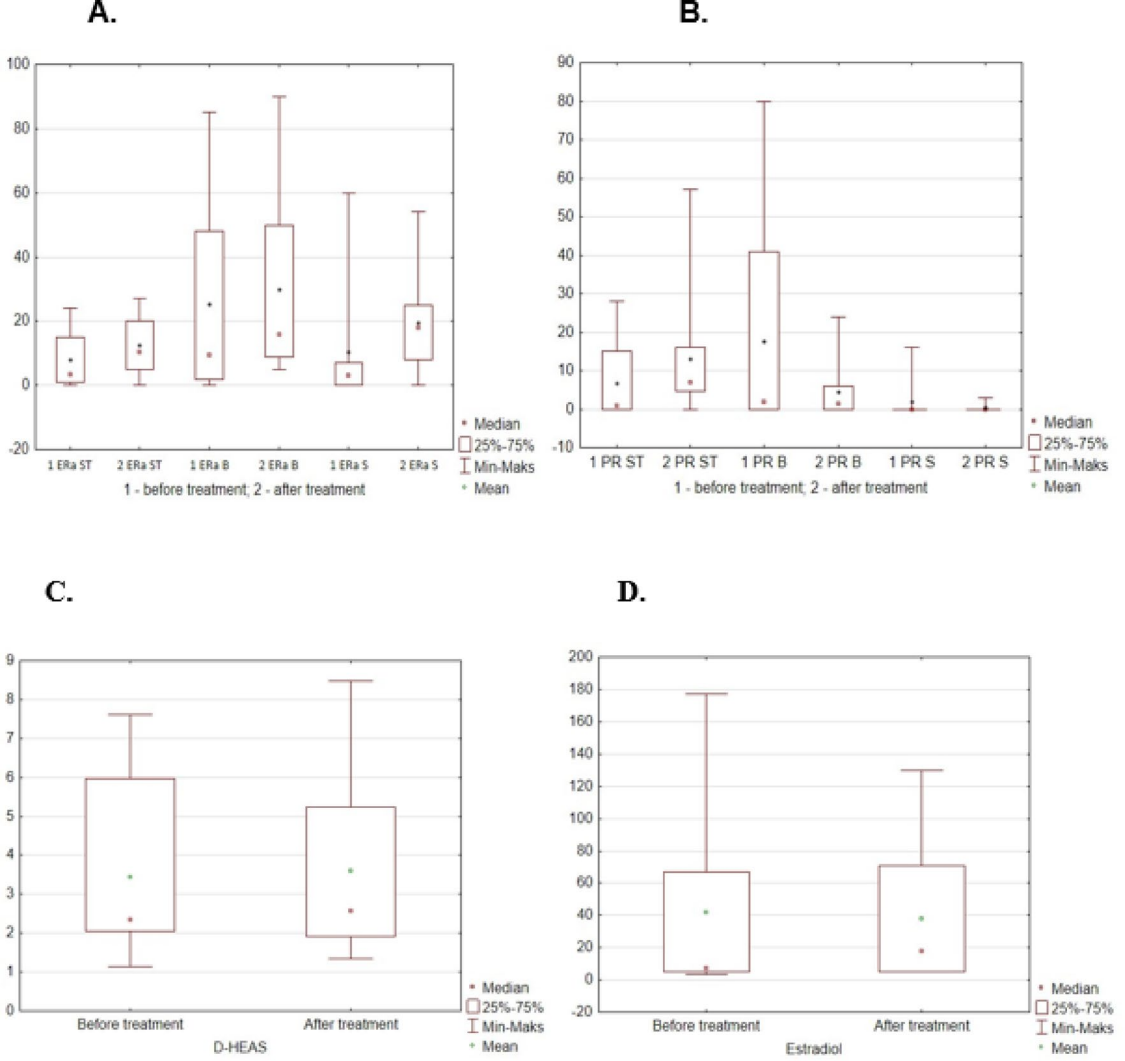
Box-plot analysis showing changes in receptor expression and hormone levels before and after 8 weeks of vaginal prasterone treatment. (**A**) Estrogen receptor alpha (ERα) expression in stroma (ST), basal layer (B), and superficial layer (S). (**B**) Progesterone receptor (PR) expression in stroma (ST), basal layer (B), and superficial layer (S). (**C**) Serum DHEA-S levels. (**D**) Serum estradiol levels. Values are presented as mean, median, interquartile range (25–75%), and minimum–maximum.

**Fig. 3:**
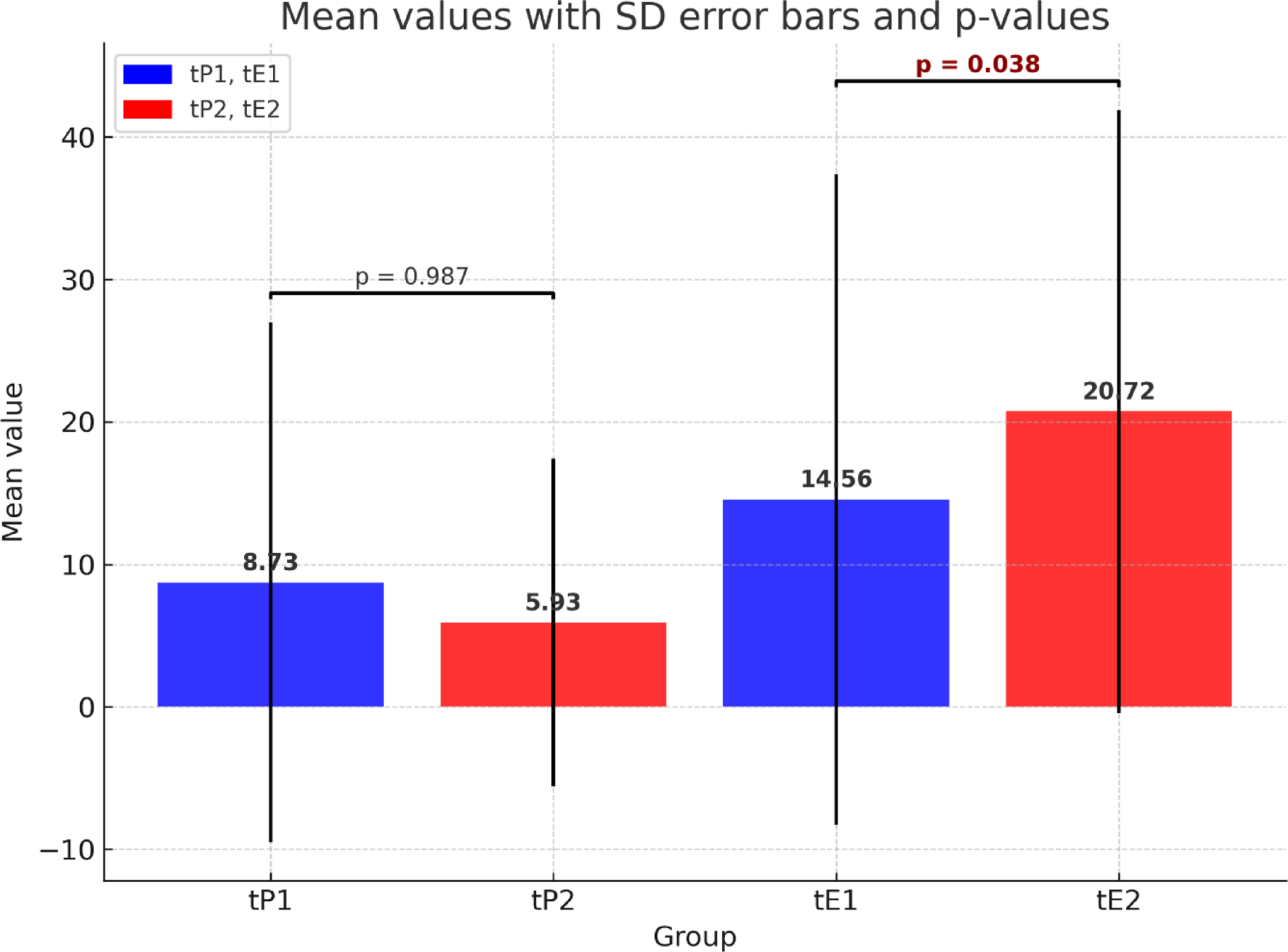
Mean values (± SD) for parameters estrogen (E) and progesterone (P) receptors in the vaginal mucosa before (1) and after (2) prasterone treatment. The values represent the mean values of receptors across the entire examined tissue. Error bars indicate standard deviations. A statistically significant increase was observed for estrogen receptors after treatment, while changes in progesterone receptors were not significant.

Figures 2 and 3 illustrate the total and individual expression levels of estrogen and progesterone receptors as well as mean value of analysed parameters.

**Fig. 4:**
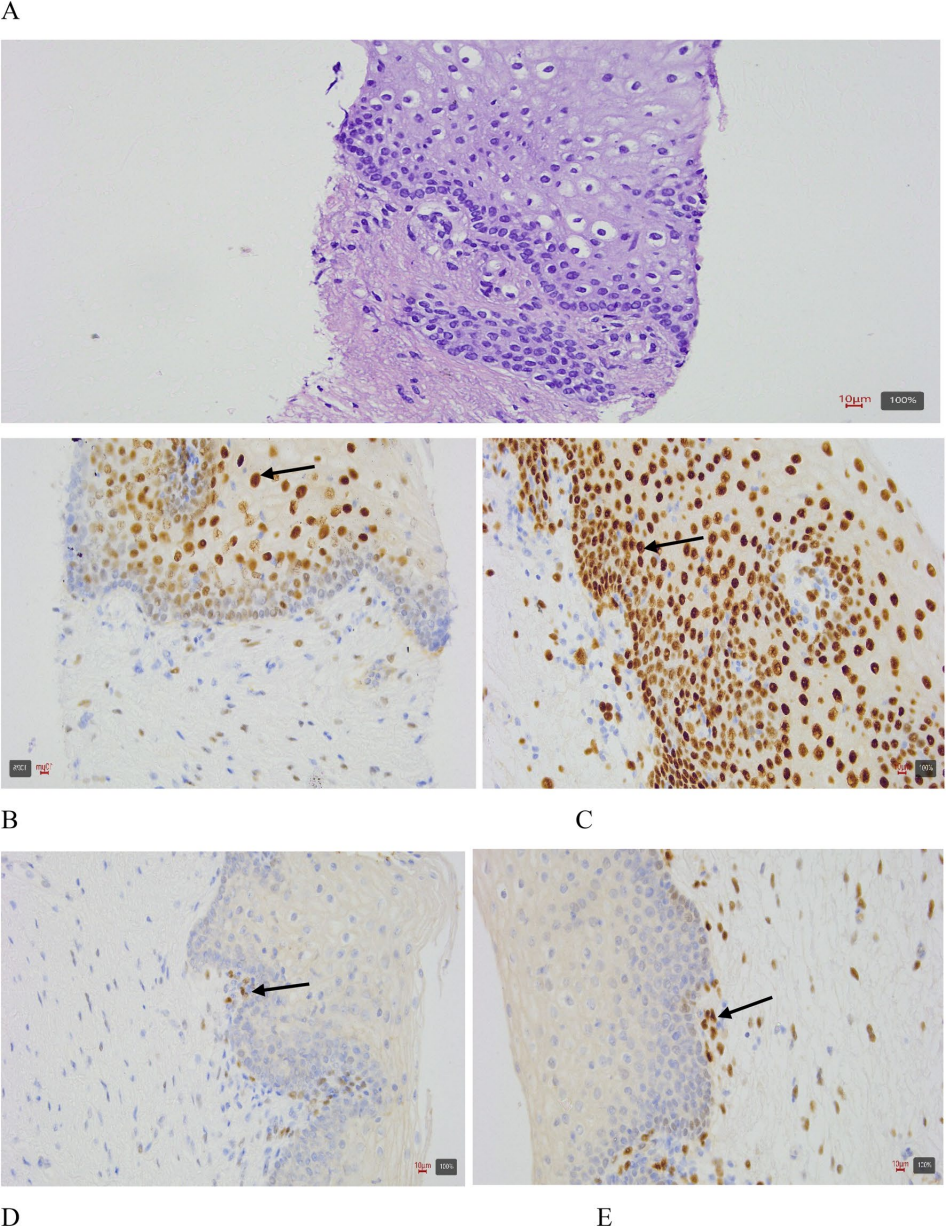
H&E and immunohistochemical staining (Autostainer Link 48 system (Dako), sample 0.2 μm) of the vaginal mucosa. (**A**) H&E; (**B**) Estrogen receptors before treatment; (**C**) Estrogen receptors after treatment; (**D**) Progesterone receptors before treatment; (**E**) Progesterone receptors after treatment. Magnification: 10×.

**Fig. 5:**
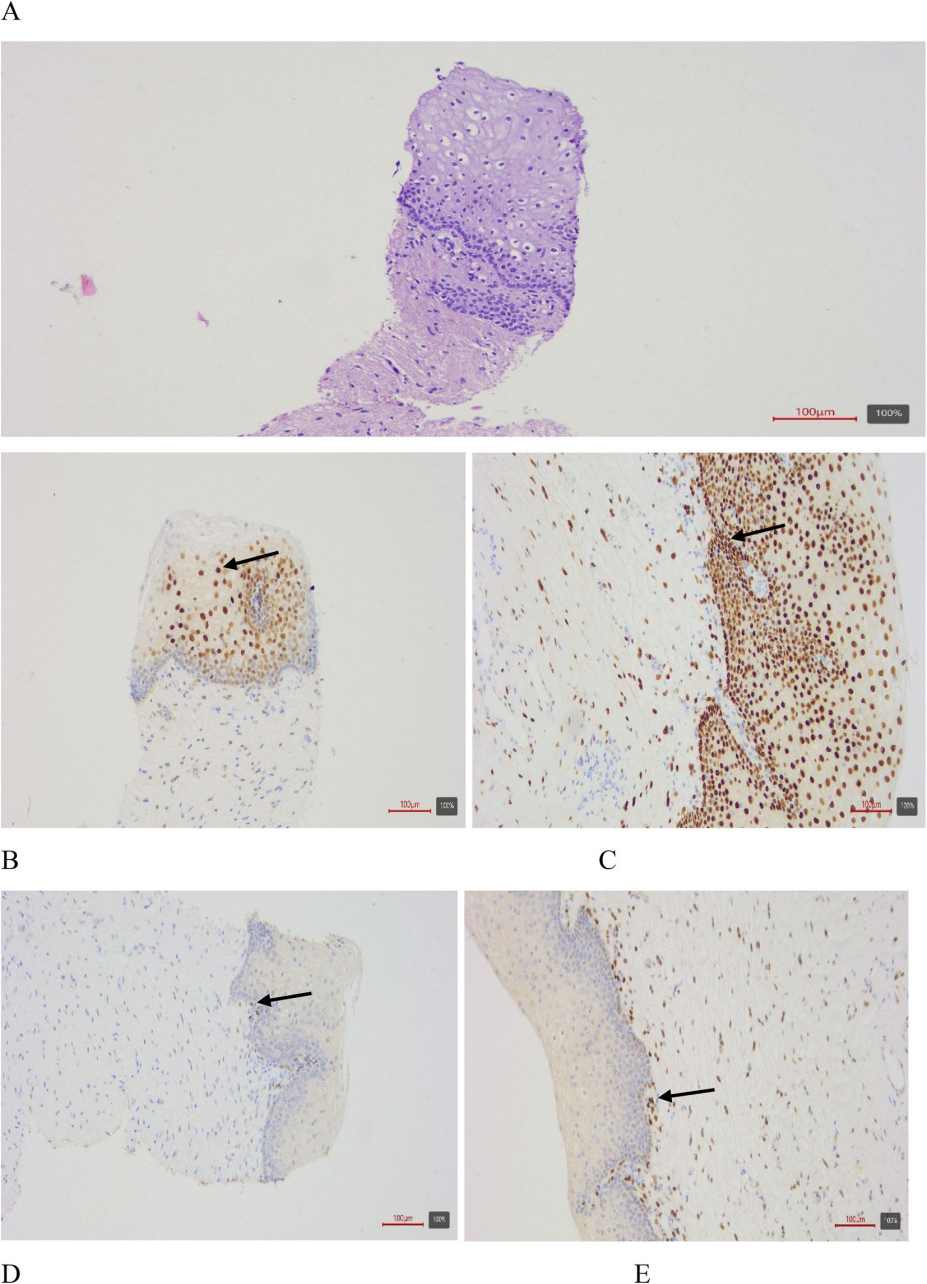
Vaginal mucosa with H&E and immunohistochemical staining (Autostainer Link 48 system (Dako), sample 0.2 μm). (**A**) H&E; (**B**) Estrogen receptors before treatment; (**C**) Estrogen receptors after treatment; (**D**) Progesterone receptors before treatment; (**E**) Progesterone receptors after treatment. Magnification: 20×.

Figures 4 and 5 illustrate the immunohistochemical staining of estrogen and progesterone receptors.

### Proteomic analysis by LC-MS/MS

LC-MS/MS analysis was used to investigate how prasterone treatment influences the molecular composition of the posterior vaginal wall, with protein abundancies before and after treatment compared with a robust linear model including patient ID as a random factor.

**Fig. 6:**
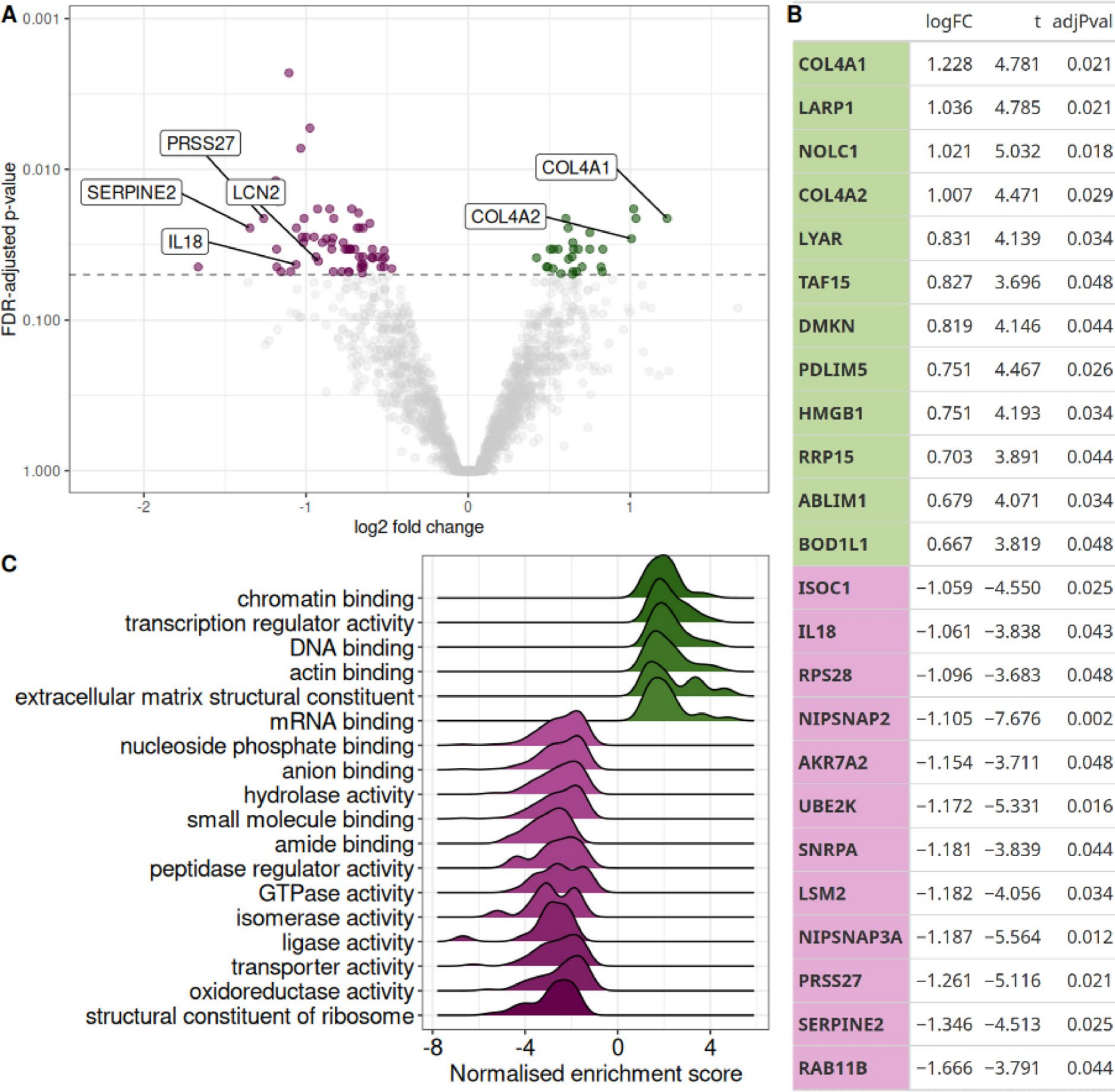
Proteomic changes in the vaginal mucosa after prasterone treatment. (**A**) Volcano plot showing differentially abundant proteins before and after treatment. Upregulated proteins (green) include extracellular matrix components such as COL4A1 and COL4A2, while downregulated proteins (purple) include inflammation-related proteins such as IL18, LCN2, SERPINE2, and PRSS27. (**B**) The ten most significantly up- and downregulated proteins are listed with log2 fold change (logFC), t-statistics, and FDR adjusted p-values (*p* < 0.05). (**C**) Gene Set Enrichment Analysis (GSEA) of Gene Ontology (GO) molecular function terms. Upregulated proteins were enriched in ECM and DNA-binding functions, whereas downregulated proteins were enriched in inflammatory and metabolic functions.

A total of 87 proteins exhibited significantly altered abundance following treatment (FDR-adjusted *p* < 0.05), as shown in Fig. 6A. A positive log2 fold change indicates upregulated proteins (*n* = 26), while a negative log2 fold change corresponds to downregulated proteins (*n* = 61). The ten most strongly up- or downregulated proteins are presented in Fig. 6B.

Gene Set Enrichment Analysis (GSEA) identified molecular functions - groups of proteins categorized by Gene Ontology - that displayed statistically significant, concordant differences between treatment conditions (Fig. 6C). From this analysis, it is clear that structural ECM proteins are generally more abundant after prasterone treatment (normalized enrichment score, NES: 1.53; FDR-adjusted *p* 0.019).

**Fig. 7:**
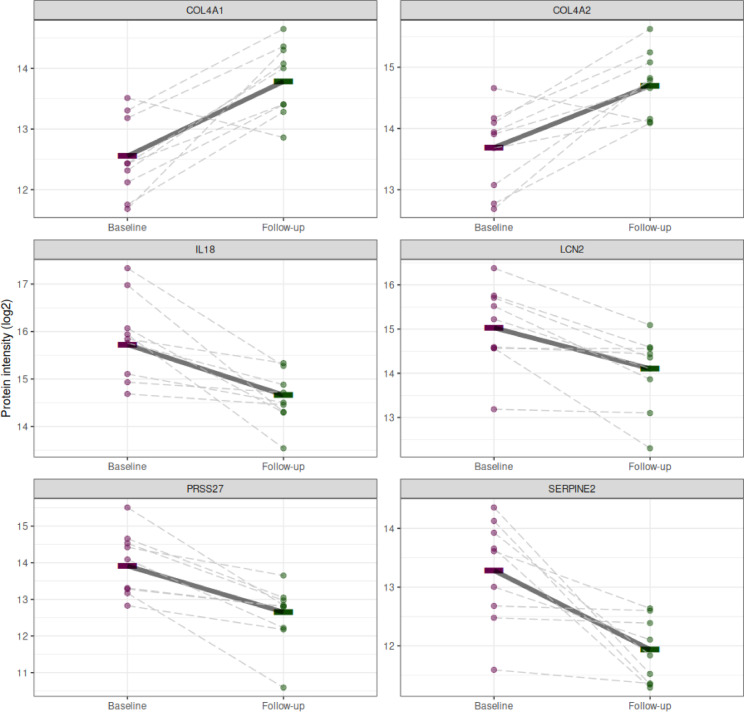
Protein abundance in vaginal mucosa before and after prasterone treatment. Protein intensities (log2) of selected targets are shown at baseline and follow-up for individual patients (dashed lines) and group means (solid lines).

Among the most upregulated proteins was collagen IV, with both the A1 and A2 chains significantly more abundant after treatment (logFC > 1; FDR-adjusted *p* < 0.05; Fig. 7). Conversely, among the most downregulated proteins were a number of inflammation-related proteins, including IL18 (interleukin-18) (logFC = − 1.06; FDR-adjusted *p* 0.047) and LCN2 (lipocalin-2) (logFC = − 0.91; FDR-adjusted *p* 0.046).

All the patients demonstrated an increased level of collagen IV A1 and A2 chains after treatment, except for one patient with the highest baseline abundance, in whom a slight decrease was observed (Fig. 7, upper panels). For IL18 and LCN2, a decrease in abundance was noted across all patients, with a more pronounced effect in those who had higher baseline levels (Fig. 7). PRSS27 and SERPINE2 also showed a decreased abundance after treatment. Together, these results highlight a shift toward enhanced structural matrix composition and reduced inflammation following prasterone therapy.

## Discussion

### Main findings

This study demonstrates that short-term vaginal prasterone therapy induces measurable molecular and structural changes in the human vaginal mucosa without altering systemic hormone levels. The increase in ERα expression across epithelial layers, together with stromal modulation of progesterone receptor expression, suggests activation of local steroid signaling pathways. These receptor changes were accompanied by epithelial thickening and upregulation of collagen IV, indicating reinforcement of the extracellular matrix and basement membrane architecture. Proteomic profiling further revealed coordinated enrichment of structural extracellular matrix proteins and downregulation of inflammation- and remodeling-related proteins, including IL18, LCN2, SERPINE2, and PRSS27. Importantly, serum estradiol and DHEA-S concentrations remained unchanged, supporting an intracrine mechanism of action in which prasterone is locally converted into active sex steroids within target tissues while minimizing systemic exposure^1,16–19^. These findings are consistent with previous clinical studies demonstrating the safety of vaginal prasterone with respect to systemic hormone levels and endometrial thickness^1,16–19^. Together, these results provide mechanistic evidence that short-term vaginal prasterone therapy promotes steroid receptor modulation and extracellular matrix remodeling in the human vaginal mucosa. Given the small sample size (*n* = 10), the study is exploratory in nature and intended to generate hypotheses that warrant confirmation in future, larger-scale studies.

### Estrogen and progesterone receptors

A significant increase in cumulative ERα expression was observed across all epithelial layers, most pronounced in the superficial layer and stroma. This pattern suggests selective modulation of receptor distribution, potentially enhancing epithelial proliferation and stromal remodeling. Similar findings have been reported in animal studies, where DHEA increased epithelial thickness and estrogen receptor signaling^20^. Mechanistic data indicate that DHEA and its metabolites may interact not only with ERα/ERβ but also with other nuclear and membrane receptors, including PXR, CAR, and GPER1^3,6,11^. Although an overall increase in ERα expression was observed, this did not reach statistical significance when individual tissue layers were analyzed separately. This may be explained by the limited sample size, which reduces statistical power, as well as by potential variability between tissue compartments. Stratification of the analysis by epithelial and stromal layers may have further reduced the ability to detect significant differences within subgroups. Therefore, these findings should be interpreted with caution and considered exploratory.

In contrast, PR expression showed divergent regulation: stromal PR was upregulated, whereas basal and superficial epithelial PR decreased. Although human data on PR after vaginal prasterone are limited, this differential response may reflect tissue-specific progesterone signaling, with stromal activation supporting remodeling and epithelial downregulation reducing proliferative turnover^6^. Stromal PR has been implicated in mediating tissue remodeling and stromal–epithelial interactions, whereas epithelial PR is more closely associated with cellular differentiation and maintenance of epithelial integrity^21,22^.

### Vaginal epithelium and extracellular matrix changes

Epithelial thickness increased after prasterone, although not statistically significant due to small sample size and variability. This trend aligns with clinical improvements in vaginal trophism reported in women treated with intravaginal DHEA for GSM^16,17^. Importantly, we demonstrated significant upregulation of collagen IV (particularly A1 and A2 chains), a novel finding in human tissue. As a key component of basement membranes, collagen IV provides structural stability^23^. Its increased abundance suggests improved biomechanical properties of the vaginal wall, potentially reducing the risk of hematomas, infections, or mesh erosions. However, these observations are speculative and should be considered hypothesis-generating, as clinical outcomes were not assessed in this study.

Nevertheless, patient-reported outcomes assessed using the WHOQOL-BREF (World Health Organization Quality of Life – BREF) questionnaire suggested a trend toward improved quality of life following treatment. The absence of statistically significant differences in WHOQOL-BREF outcomes may be explained by both the limited sample size and the relatively short duration of the intervention (8 weeks), as changes in quality-of-life measures typically require longer follow-up periods to become statistically detectable.

Interestingly, while systemic DHEA administration in animal models has been associated with fibrotic changes mediated by TGF-β signaling^24^, our results do not support a generalized profibrotic effect. Instead, we observed selective upregulation of basement membrane collagen IV following local administration, which may reflect targeted tissue remodeling and structural reinforcement rather than fibrosis.Proteomic analysis and immune regulation.

Proteomic analysis revealed 87 proteins with significantly altered abundance. Structural ECM proteins were enriched, whereas several inflammation- and remodeling-related proteins (IL18, LCN2, SERPINE2, PRSS27) were downregulated. IL18 and LCN2 are mediators of innate immunity and antimicrobial defense^25–27^; their reduction may attenuate inflammatory signaling and oxidative stress, supporting a more quiescent mucosal environment. Decreased SERPINE2 and PRSS27 expression indicates a shift toward ECM stabilization and epithelial differentiation^28–32^. To our knowledge, such coordinated downregulation has not previously been reported in vaginal prasterone treatment in humans. While potentially advantageous for surgical preparation, possible trade-offs, including altered antimicrobial responsiveness, require further study.

**Fig. 8:**
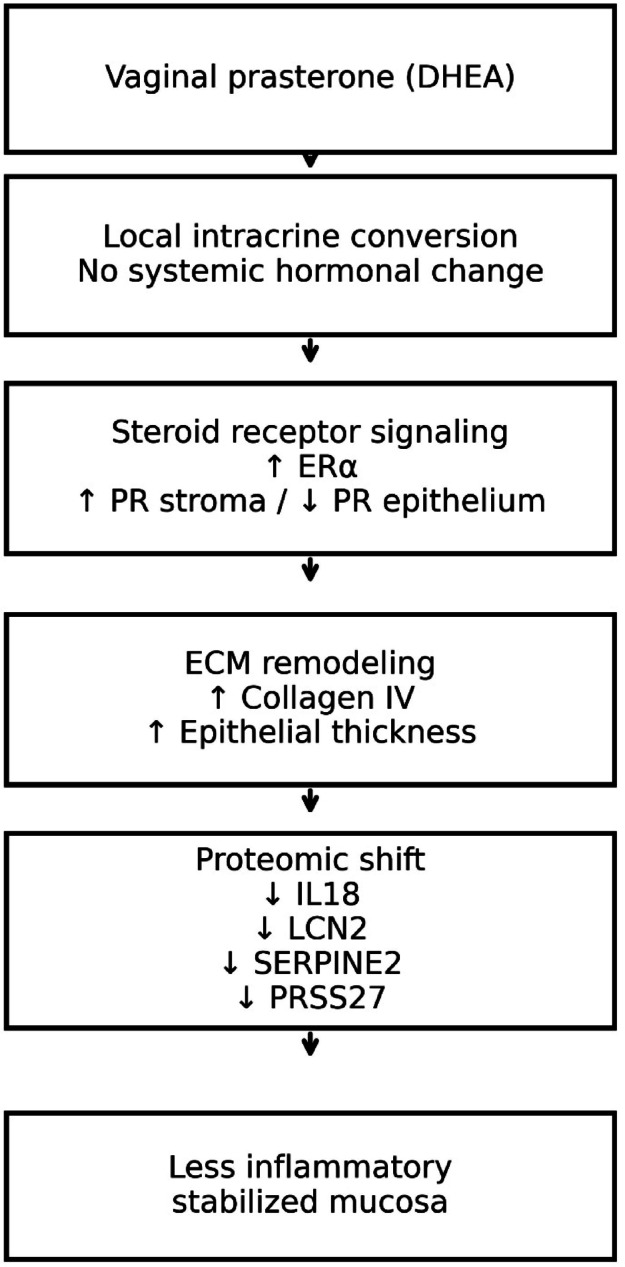
Proposed mechanism of vaginal prasterone action in human vaginal mucosa. Prasterone undergoes local intracrine conversion in vaginal tissue, leading to modulation of steroid receptor signaling, extracellular matrix remodeling, and proteomic changes consistent with a less inflammatory and structurally stabilized mucosa.

Based on the findings of this study, the potential mechanism of vaginal prasterone action in the vaginal mucosa is illustrated in Fig. 8.

### Strengths and limitations

Overall, prasterone appears to exert dual effects: (1) strengthening the vaginal wall through increased ERα expression, epithelial thickness, and collagen IV deposition; and (2) shifting the mucosal microenvironment toward a less inflammatory and more structurally consolidated phenotype. This combination may be advantageous in preoperative urogynecology, where tissue quality is critical for surgical success. These findings align with previous clinical and biochemical evidence supporting DHEA in GSM, osteoporosis, and hypoactive sexual desire disorder^16–19^, while extending current knowledge through in-depth proteomic analysis demonstrating ECM reinforcement and immunomodulation.

Although several parameters did not reach statistical significance, consistent trends—including increased ERα expression and epithelial thickening, and reduced epithelial PR—indicate a measurable local effect. The statistically significant rise in cumulative ERα further supports this conclusion. These molecular and structural changes, though subtle, may have biological relevance.

Limitations include the small cohort size and short treatment duration, limiting generalizability. Larger, long-term studies are required to confirm durability, safety, and clinical benefits in symptomatic treatment and preoperative settings. Although the cohort included both peri- and postmenopausal women, hormonal status was carefully characterized, and results remained consistent after excluding perimenopausal participants. The heterogeneity of the study population, including both perimenopausal and postmenopausal women as well as patients with different urogynecological disorders, was intentional and reflected the clinical objective of assessing the effect of prasterone on vaginal tissues in women presenting with urogynecological dysfunction. At the same time, such heterogeneity may have influenced baseline tissue characteristics and treatment response.

The findings should be interpreted with caution and considered exploratory. Future studies conducted in larger and more homogeneous subgroups are warranted to determine whether menopausal status or the type of urogynecological disorder affects tissue response to prasterone.

Interpretation (in light of other evidence)

Our findings are consistent with clinical trials showing minimal systemic hormonal exposure during vaginal prasterone therapy^1^. However, unlike large randomized studies focused on symptom relief and safety, our investigation explored molecular mechanisms in a small exploratory cohort and should be considered hypothesis-generating.

Experimental data demonstrate that DHEA enhances estrogen receptor signaling and epithelial proliferation, supporting the observed ERα upregulation. However, receptor expression does not necessarily reflect receptor activation or downstream transcriptional activity; functional studies are needed to confirm enhanced signaling.

The increase in collagen IV suggests basement membrane reinforcement, yet whether this translates into measurable biomechanical improvements or better surgical outcomes remains unclear. Similarly, downregulation of inflammatory and remodeling proteins may indicate reduced inflammatory signaling but could also reflect altered immune responsiveness with uncertain implications.

Compared with vaginal estradiol, the absence of systemic hormonal exposure with prasterone may represent a potential safety advantage, particularly in women with contraindications to systemic estrogen therapy^4^. However, this assumption remains theoretical, as direct comparative mechanistic studies are lacking, and no conclusions regarding equivalence or superiority can be drawn.

In summary, short-term vaginal prasterone induces measurable local molecular changes consistent with mucosal remodeling. The extent to which these alterations translate into clinically meaningful benefits—particularly in preoperative urogynecology preparation—requires confirmation in larger randomized studies incorporating functional and clinical endpoints.

## Conclusions

1. Short-term (8 weeks) vaginal prasterone significantly increased ERα expression across all epithelial layers, with cumulative expression reaching statistical significance.
2. Collagen IV (A1 and A2 chains) abundance and epithelial thickness increased, suggesting improved vaginal wall biomechanics.
3. Progesterone receptors showed differential regulation: stromal upregulation with epithelial downregulation.
4. Proteomic analysis demonstrated downregulation of inflammation- and remodeling-related proteins (IL18, LCN2, SERPINE2, PRSS27), indicating a shift toward a less inflammatory and more structurally stabilized mucosa.
5. Serum estradiol and DHEA-S levels remained unchanged, confirming a local (intracrine) mechanism without systemic hormonal effects.
6. Vaginal prasterone may have implications for tissue quality and vaginal mucosal remodeling; however, larger long-term studies are needed to confirm safety and durability.
7. Short-term vaginal prasterone therapy was associated with a non-significant trend toward improved quality of life.

## Supplementary Information

Below is the link to the electronic supplementary material.

Supplementary Material 1

## Data Availability

The datasets used and/or analysed during the current study available from the corresponding author on reasonable request.
