# Supplementary material for: Short-term vaginal prasterone therapy induces steroid receptor modulation and extracellular matrix remodeling in human vaginal mucosa
Source: Sci Rep. 2026 May 19;16:22625. doi: 10.1038/s41598-026-50491-5 (PMC13381653; doi:10.1038/s41598-026-50491-5)
Supplement: Supplementary file 1 — Supplementary Material 1 [file 41598_2026_50491_MOESM1_ESM.docx]

| n | Estradiol  [pg/ml] | Estradiol1  [pg/ml] | DHEAS  [µg/dl] | DHEAS1  [µg/dl] | ER1α ST | ER1α B | ER1α S | ER2α ST | ER2α B | ER2α S | P1 ST | P1 B | P1 S | P2 ST | P2 B | P2 S | Epitelial tickness1 [mm] | Epitelial tickness2 [mm] |
| --- | --- | --- | --- | --- | --- | --- | --- | --- | --- | --- | --- | --- | --- | --- | --- | --- | --- | --- |
| I | 5 | 5 | 2,9 | 2,9 | 9 | 19 | 5 | 5 | 22 | 24 | 15 | 41 | 2 | 8 | 0 | 0 | 130 | 120 |
| II | 3,6 | 5 | 1,5 | 1,4 | 2,5 | 0 | 4,3 | 17,5 | 18 | 54 | 0 | 0 | 0 | 4,7 | 6 | 0 | 70 | 90 |
| III | 177,2 | 75,6 | 7,6 | 8,5 | 15 | 48 | 0,03 | 24 | 73 | 25 | 28 | 47 | 0 | 57 | 24 | 0,07 | 90 | 160 |
| IV | 66,9 | 129,6 | 6,3 | 5,3 | 1 | 0 | 0 | 2 | 5 | 0 | 0 | 0 | 0 | 2 | 2 | 0 | 17 | 26 |
| V | 5 | 5 | 1,1 | 1,9 | 0 | 9 | 2 | 10 | 13 | 20 | 0 | 4 | 16 | 6 | 1 | 0 | 50 | 83 |
| VI | 5 | 5 | 2,3 | 2,6 | 1 | 4 | 0 | 27 | 5 | 36 | 0 | 0 | 0 | 16 | 0 | 0 | 54 | 68 |
| VII | 10 | 19,3 | 2,3 | 2,6 | 23 | 75 | 60 | 0 | 9 | 0 | 19 | 80 | 0 | 0 | 9 | 0 | 75 | 55 |
| VIII | 5 | 18,2 | 2,4 | 1,8 | 24 | 85 | 25 | 20 | 50 | 16 | 2 | 0 | 0 | 10 | 0 | 0 | 90 | 60 |
| IX | 126,4 | 71,1 | 6 | 6,3 | 0 | 2 | 7 | 11 | 14 | 8 | 0 | 0 | 0 | 20 | 3 | 3 | 20 | 90 |
| X | 13,9 | 13,8 | 2,1 | 2,1 | 4 | 10 | 2 | 10 | 90 | 13 | 4 | 4 | 0 | 6 | 0 | 0 | 50 | 60 |

**Table 1.** Values of serum estradiol and DHEA-S levels, mean percentage of ERα and PR receptor expression and mean epithelial thickness

before (1) and after (2) vaginal prasterone treatment.

ERα B – ERα in the basal layer, ERα S – ERα in the superficial layer, ERα ST – ERα in the stroma, PR B – PR in the basal layer, PR S – PR in the superficial layer, PR ST – PR in the stroma
